# Revealing An Intercalation‐Conversion‐Heterogeneity Hybrid Lithium‐Ion Storage Mechanism in Transition Metal Nitrides Electrodes with Jointly Fast Charging Capability and High Energy Output

**DOI:** 10.1002/advs.202203895

**Published:** 2022-10-06

**Authors:** Fei Li, Yadong Li, Linyi Zhao, Jie Liu, Fengkai Zuo, Fangchao Gu, Hengjun Liu, Renbin Liu, Yuhao Li, Jiqiang Zhan, Qiang Li, Hongsen Li

**Affiliations:** ^1^ College of Physics Center for Marine Observation and Communications Qingdao University Qingdao 266071 China

**Keywords:** energy storage mechanism, fast charging, high energy density, lithium‐ion capacitors

## Abstract

The performance of electrode materials depends intensively on the lithium (Li)‐ion storage mechanisms correlating ultimately with the Coulombic efficiency, reversible capacity, and morphology variation of electrode material upon cycling. Transition metal nitrides anode materials have exhibited high‐energy density and superior rate capability; however, the intrinsic mechanism is largely unexplored and still unclear. Here, a typical 3D porous Fe_2_N micro‐coral anode is prepared and, an intercalation–conversion–heterogeneity hybrid Li‐ion storage mechanism that is beyond the conventional intercalation or conversion reaction is revealed through various characterization techniques and thermodynamic analysis. Interestingly, using advanced in situ magnetometry, the ratio (ca. 24.4%) of the part where conversion reaction occurs to the entire Fe_2_N can further be quantified. By rationally constructing a Li‐ion capacitor comprising 3D porous Fe_2_N micro‐corals anode and commercial AC cathode, the hybrid full device delivers a high energy‐density (157 Wh kg^−1^) and high power‐density (20 000 W kg^−1^), as well as outstanding cycling stability (93.5% capacitance retention after 5000 cycles). This research provides an original and insightful method to confirm the reaction mechanism of material related to transition metals and a fundamental basis for emerging fast charging electrode materials to be efficiently explored for a next‐generation battery.

## Graphical abstract

Combining various characterization techniques and thermodynamic analysis, the Li‐ion storage process in transition metal nitrides (TMNs) is reacquainted, showing an intercalation–conversion–heterogeneity hybrid mechanism, which may be one of the reasons for the fast charging and high energy output of TMNs electrode materials.

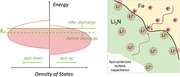

## 1 Introduction

As the ever‐increasing applications of the lithium‐ion batteries (LIBs), from cell phones and portable electronics to electric vehicles and electric grids, the demands on batteries with both high‐energy density and fast‐charge capability are continuously increasing.^[^ ^1^, ^2^, ^3^, ^4^ ^]^ In the past decade, a landmark process has been achieved in promoting the electrochemical performance by engineering of the battery structure, employing solid‐state electrolyte, and optimizing electrode design.^[^ ^5^, ^6^ ^]^ However, current high‐energy density devices batteries are unable to achieve fast charging without detrimentally impacting battery performance and safety.^[^ ^7^, ^8^, ^9^ ^]^ When LIBs are charged at high rates, increased cell polarization results in limited energy utilization, increased capacity fade, excessive heat generation and other deleterious effects, which severely limit the charging time of the state‐of‐the‐art device batteries.^[^ ^10^ ^]^ Therefore, there is an unmet need to develop LIBs that can synchronously achieve high‐energy density and high‐efficiency fast charging.^[^ ^11^, ^12^, ^13^ ^]^ To address these technological challenges, an in‐depth understanding of the Li‐ion storage mechanism within the electrodes is extremely desired.

Transition metal nitrides (TMNs), with high electronic conductivity and good ionic diffusion arising from the vacancies within their crystal structures, have shown promising performance for various applications in energy storage devices.^[^ ^14^ ^]^ For instance, Liu et al.^[^ ^15^ ^]^ prepared a micrometer‐sized porous Fe_2_N/C bulk anode material that exhibits exceptional charge/discharge rate performance for LIBs. In particular, as the current density increased from 0.1 to 6.0 A g^−1^, the capacity retention was as high as 40%. Moreover, Li et al.^[^ ^16^ ^]^ applied TMNs materials to the field of supercapacitors, with CoN‐Ni_3_N/N‐C/CC as the cathode and VN/CC as the anode, showing an outstanding energy density of 106 µWh cm^−2^ and maximum power density of 40 mW cm^−2^. Due to the excellent rate capability of the NbN@C composite anode in Li‐ion half‐cells, Zhou et al.^[^ ^17^ ^]^ used it in hybrid ion capacitors to match the activated carbon cathode with high power characteristics, which can deliver a high energy density of 53.8 Wh kg^−1^ at a high power density of 7818 W kg^−1^. According to these studies, there is widespread consensus that TMN has advantages in high‐energy density and fast‐charging capability, and the reason is simply attributed to the good electrical conductivity of TMNs, but the intrinsic mechanism is rarely reported and still unclear. We admit that it is difficult for these materials to be applied in practical applications at present, nonetheless, the research on their reaction mechanism is very interesting, which can provide a reasonable explanation to solve the above dilemma.

Herein, we report a fundamental investigation on the Li‐ion storage mechanism of a typical Fe_2_N anode using a series of experimental characterizations and thermodynamic analysis. The as‐synthesized 3D porous Fe_2_N micro‐coral electrode materials show a reversible capacity of 350 mAh g^−1^ at 0.5 A g^−1^. When the current density reaches 5.0 A g^−1^, the capacity retention can be maintained at as high as ≈50%, indicating high reversibility and excellent rate capability. A suite of in‐depth characterizations encompassing in situ magnetometry, in situ X‐ray diffraction (XRD), ex situ X‐ray photoelectron spectroscopy (XPS), ex situ transmission electron microscopy (TEM) and thermodynamic analysis suggest that the lithium storage in this material occurs with an intercalation–conversion–heterogeneity hybrid mechanism. Furthermore, the ratio (ca. 24.4%) of the part where conversion reaction occurs to the entire Fe_2_N material can be quantified by calculating the variation in magnetism. We further fabricated a Li‐ion hybrid capacitor (LIC) by utilizing this 3D porous Fe_2_N micro‐corals as anode together with activated carbon (AC) cathode, which delivered an outstanding power density of 4000 W kg^−1^ at a high energy density of 100.7 Wh kg^−1^ and excellent capacity retention property even after 5000 cycles.

## 2 Result and Discussion

**Figure 1:**
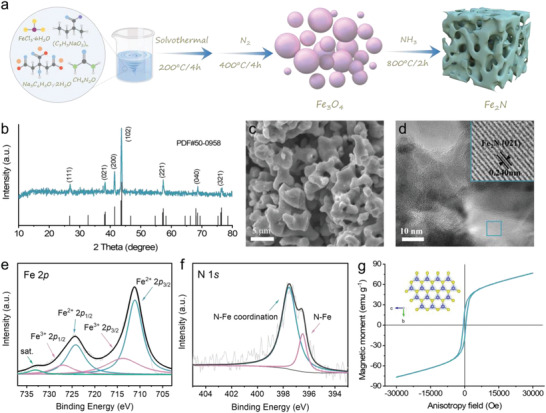
Synthetic process and characterization of 3D porous Fe_2_N micro‐corals. a) Schematic illustration of the synthesis of 3D porous Fe_2_N micro‐corals. b) XRD pattern, c) SEM image, and d) HRTEM image of 3D porous Fe_2_N micro‐corals. High‐resolution XPS spectra of e) Fe 2*p* and f) N 1*s*. g) MH curve of 3D porous Fe_2_N micro‐corals.

The overall synthesis strategy for 3D porous Fe_2_N micro‐corals is schematically illustrated in **Figure** **1**a. Initially, a certain amount of raw materials, including ferric chloride hexahydrate and sodium citrate dihydrate, urea and sodium polyacrylate, were first dissolved in deionized water to form a homogeneous solution. Subsequently, the solution mentioned above underwent a hydrothermal process (200 °C, 4 h) and then dried to a powder followed by a direct thermal treatment (400 °C, 4 h) in Ar atmosphere to obtain Fe_3_O_4_ nanospheres. Finally, the 3D porous Fe_2_N micro‐corals were obtained via further being subject to annealing (800 °C, 2 h) under a NH_3_ atmosphere.

The crystal structure of as‐synthesized Fe_2_N was studied by XRD, as denoted in Figure 1b, and the characteristic peaks could be completely consistent with the Fe_2_N (PDF#50‐0958) standard card. The morphologies and microstructures were characterized by scanning electron microscopy (SEM) and TEM. As shown in Figure S1, Supporting Information, the precursor material Fe_3_O_4_ grows uniformly in the form of nanosphere morphology. After annealing, the as‐prepared material Fe_2_N exhibits a 3D porous micro‐coral structure (Figure 1c and Figure S2, Supporting Information) which can provide a highly exposed surface owing to its good contact with the electrolyte. Under close observation from high‐resolution transmission electron microscopy (HRTEM, Figure 1d) image, the lattice fringes with spacing of 0.24 nm can be observed, which is in good agreement with the interplanar spacing of the Fe_2_N planes. Additionally, the elemental composition and bonding characteristics of 3D porous Fe_2_N micro‐corals were further confirmed with XPS technique. As shown in Figure S3, Supporting Information, typical Fe, N, O and C signals were detected in Fe_2_N samples. In the high‐resolution Fe 2*p* spectrum (Figure 1e), the deconvoluted peaks at 711.1 and 724.2 eV can be ascribed to Fe 2*p*_3/2_ and Fe 2*p*_1/2_ orbitals of Fe^2+^ species and the peaks at 713.8 and 727.0 eV correspond to Fe 2*p*_3/2_ and Fe 2*p*_1/2_ orbitals of Fe^3+^ species, together with a satellite peak at a high binding energy of 733.1 eV.^[^ ^18^ ^]^ The N 1*s* spectrum (Figure 1f) can be deconvoluted into two peaks located at 396.4 and 397.7 eV, corresponding to N—Fe and N—Fe coordination compounds, respectively.^[^ ^19^ ^]^ All these results indicate the successful synthesis of 3D porous Fe_2_N micro‐corals, which is consistent with the above XRD and HRTEM results. It is noted that observing the magnetization hysteresis (MH) curve for a material provides a glimpse into its magnetic properties. As shown in Figure 1g, MH curve demonstrates that the prepared Fe_2_N samples exhibit ferromagnetism with some high magnetization of 76.8 emu g^−1^ at a magnetic field of 3T.

**Figure 2:**
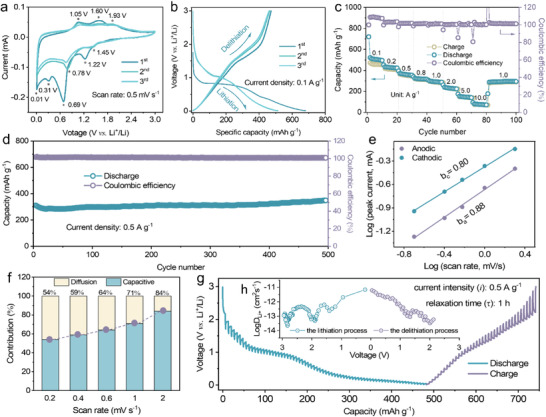
Electrochemical analysis of Fe_2_N micro‐coral electrodes in half‐cells. a) Typical CV curves at a scan rate of 0.5 mV s^−1^. b) GCD profiles for the first 3 cycles. c) Rate capabilities at different current densities. d) Long‐term cycle performance at a current density of 0.5 A g^−1^. e) Determination of the *b* value using the relationship between peak current and scan rate. f) Normalized contribution ratio of capacitive and diffusion at different scan rates. g) GITT voltage profiles and h) diffusion coefficients versus state of charge and discharge.

To investigate the Li‐storage behaviors of the Fe_2_N electrodes, cyclic voltammetry (CV), galvanostatic charge–discharge (GCD), rate performance and cycling durability tests were conducted by assembling the half‐cells with metallic lithium serving as counter electrodes. **Figure** **2**a presents the initial three CV curves of Fe_2_N electrode in a potential range of 0.01–3.0 V at a scanning rate of 0.5 mV s^−1^. During the first cathodic scan, three reduction peaks appear at ≈1.22, ≈0.69 and ≈0.31 V and disappear in the subsequent cycles, on account of the irreversible side reactions and the formation of stable solid‐electrolyte‐interphase (SEI) film on the surface of electrode.^[^ ^20^, ^21^ ^]^ As for the anodic scan, two oxidation peaks occur at 1.05 and 1.60 V, which can be associated to the extraction of Li^+^ from Fe_2_N electrode. Apparently, the CV curves have a similar shape from the second cycle onward, implying that the Fe_2_N electrode exhibits good stability and reversibility during the Li^+^ insertion/extraction. According to the previous reports, two pairs of redox peaks are assigned to the two stages of Li^+^ insertion/extraction and conversion reactions. In detail, the peak at 1.45 V corresponds to the insertion of Li^+^ into the interlayers of Fe_2_N to form Li*_x_* Fe_2_N, and the peak at 0.78 V can be attributed to the conversion of Li*_x_* Fe_2_N to Fe^0^ and Li_3_N.^[^ ^21^ ^]^ Figure 2b shows the GCD profiles for the Fe_2_N electrode at a current density of 0.1 A g^−1^. Conspicuously, the curves of the second and third cycles almost coincide with each other, indicating the good cycle stability, which is consistent with CV measurement results. In addition, the rate performance of Fe_2_N electrode was further evaluated for step‐wise increased current densities from 0.1 to 10 A g^−1^ (Figure 2c). The specific capacities are 504, 428, 361, 320, 293, 232, 147 and 77 mAh g^−1^ at the current densities of 0.1, 0.2, 0.5, 0.8, 1.0, 2.0, 5.0 and 10.0 A g^−1^, correspondingly. Notably, the capacity can be almost fully recovered as the current density returns back to the 1.0 A g^−1^ and the Coulombic efficiency is close to 100%, demonstrating good electrochemical reversibility. The long‐term cycling performance of Fe_2_N electrodes at high current densities was also investigated. At 0.5 A g^−1^, the Fe_2_N electrode can obtain a reversible capacity of 350 mAh g^−1^ after 500 cycles, indicating excellent cycling stability (Figure 2d). Meanwhile, the excellent electrochemical performance of Fe_2_N electrode can be supported by electrochemical impedance spectroscopy (EIS) test. Figure S4, Supporting Information, displays the Nyquist plots of the samples, which are characterized by a depressed semicircle at high/medium frequency and an inclined line at low frequency.^[^ ^22^ ^]^ It turns out that the Fe_2_N exhibits the relatively small charge transfer resistance (*R*_ct_) and slope of straight line at the fully discharged state of both the 10th and 100th cycles, verifying the rapid Li^+^ transportation kinetics for this material. Additionally, the *R*_ct_ of Fe_2_N shows slight change with cycling, while the electrolyte‐diffusion resistances (*R*_e_) almost unchanged at low frequency region (see Table S1, Supporting Information), suggesting the stable of the electrode material and efficient ion transport.^[^ ^23^, ^24^ ^]^

CV measurement also provides great insight into the Li‐ion storage behavior and electrochemical reaction kinetics of the electrode. As shown in Figure S5, Supporting Information, the CV curves of Fe_2_N electrode have similar shapes at various scan rates ranging from 0.2 to 2.0 mV s^−1^, where the measured peak current (*i*) and scan rate (*ν*) obey the following power‐law equation:^[^ ^25^, ^26^, ^27^ ^]^

(1) $$i=a\nu^{b}$$

where *b* reveals the charge storage behaviour and can be calculated from the slope of log(*i*) against log(*ν*) plot. In general, the *b* value of 0.5 represents a diffusion‐controlled process, whereas the *b* value of 1.0 indicates a surface capacitive‐dominated effect. As shown in Figure 2e, the *b* value of the cathodic and anodic peaks is calculated to be 0.80 and 0.88, respectively, implying a major pseudocapacitive‐controlled process for Li‐ion storage of Fe_2_N anode. In such context, the proportion of the above two behaviors under different scan rates can be further quantified by the equation:^[^ ^28^, ^29^ ^]^

(2) $$i\left(V\right)=k_{1}\nu +k_{2}\nu^{1/2}$$

where *k*_1_ *ν* and *k*_2_ *ν* ^1/2^ correspond to the contribution from pseudocapacitive effect and diffusion‐controlled process, respectively. As representatively displayed in Figure S6, Supporting Information, the green color‐shaded region corresponds to the capacitive current response out of the total at the scan rate of 0.5 mV s^−1^, accounting for a pseudocapacitive fraction of ≈71%. As such, the fractions of the capacitive contribution at various scan rates are summarized in Figure 2f, when the scan rate increases, the capacitive contribution further increases from 54% to 84%. Moreover, galvanostatic intermittent titration technique (GITT) was carried out in a coin cell to evaluate the diffusion coefficient of Li^+^ ($D_{{\mathrm{Li}}^{+}}$) in the Fe_2_N electrode after it was given 20 cycles to reach its thermal equilibrium state. Figure 2g shows a series of pulse current at 0.5 A g^−1^ for 10 min and the time interval between adjacent pulses for 1 h during a charge and discharge process. The $D_{{\mathrm{Li}}^{+}}$ can be calculated according to the simplified Fick's second law with the following equation:^[^ ^30^, ^31^, ^32^ ^]^

(3) $$D=\frac{4}{\pi \tau }{\left(\frac{m_{B}V_{M}}{M_{B}S}\right)}^{2}{\left(\frac{\Delta E_{s}}{\Delta E_{\tau }}\right)}^{2}$$

where *τ* is relaxation time for each discharge process, *m*_B_, *M*_B_ and *V*_M_ denote the active mass, molar mass and molar volume of electrode material, *S* is the interfacial area between the electrode and the electrolyte, Δ*E_s_* and Δ*E*_*τ*_ is defined as the steady‐state voltage change due to the current pulse and the voltage change during the constant current pulse regardless of the *IR* drop (Figure S7, Supporting Information). The calculated values of $D_{{\mathrm{Li}}^{+}}$ for the Fe_2_N electrode fall in between 10^−13.6^ and 10^−11.2^ cm^2^ s^−1^, indicating the fast Li‐ions diffusion during lithiation and delithiation process. This excellent electrochemical performance may be partly attributed to the unique structure. The porous structure is effective in mitigating the swelling and maintaining structural integrity, and facilitates the efficient penetration of the electrolyte into the structure, thereby facilitating electron transfer kinetics.^[^ ^33^ ^]^

**Figure 3:**
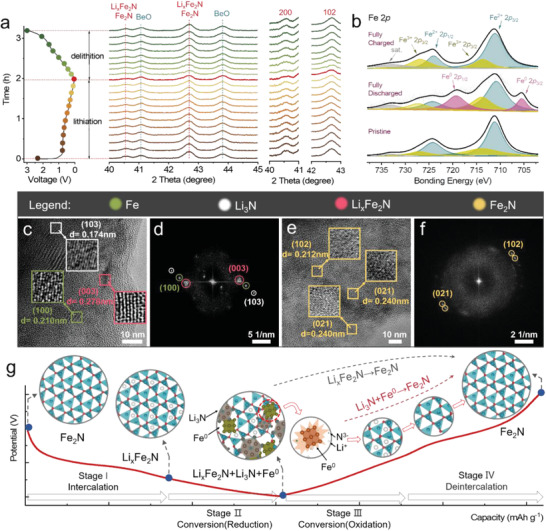
In situ*/*ex situ observation of the phase transformation. a) The galvanostatic curves of the Fe_2_N electrodes (left) and in situ XRD patterns for the first lithiation and delithiation processes (right). b) Ex situ high resolution XPS spectra of Fe 2*p* on the surface of Fe_2_N electrodes at different discharge/charge states. Ex situ HRTEM images and FFT patterns of Fe_2_N electrodes after discharging to 0.01 V (c,d) and after charging to 3.0 V (e,f). g) Schematic illustration for the lithium storage reaction mechanism of Fe_2_N anode.

To thoroughly track the phase evolution and probe into the intrinsic Li‐ion storage mechanism of the Fe_2_N anode, in situ XRD, ex situ XPS, HRTEM and fast Fourier transform (FFT) characterizations were applied to systematically depict the lithiation/delithiation process. **Figure** **3**a describes the obtained in situ XRD test with a sampling interval of 5 min was implemented for the 1st cycle at 0.2 A g^−1^. When the Fe_2_N electrode was discharged from open‐circuit potential to 0.01 V, the predominant peaks at 40.5° (200) and 42.7° (102) gradually weaken but do not disappear completely, which corresponds to the generation of Li*_x_* Fe_2_N. At the fully charged state, peaks of Fe_2_N appear again, which means the Li‐ions by charging process could be released to the external and exhibit excellent reversibility in the cycle and response for the stable cycle performance. Strikingly, no new peaks beyond Li*_x_* Fe_2_N structure were observed during the cycling, indicating the Li‐ion storage in Fe_2_N electrode is either a complete intercalation process or only partial conversion which cannot be identified via in situ XRD. In previous reports, researchers found that the conversion products of Fe_2_N anode are Fe^0^ and Li_3_N, however, no pronounced characteristic diffraction peaks of these species can be observed during the cycling. To examine this issue, ex situ XPS was applied to analyze the changes of Fe 2*p* signals at different voltage points. After the Fe_2_N anode is fully discharged, the peaks at 710.8, 713.9, 724.4, 727.6, 705.4 and 719.2 eV related to Fe^2+^ 2*p*_3/2_, Fe^3+^ 2*p*_3/2_, Fe^2+^ 2*p*_1/2_, Fe^3+^ 2*p*_1/2_, Fe^0^ 2*p*_3/2_ and Fe^0^ 2*p*_1/2_ signal are observed in Figure 3b, which could be assigned to Li*_x_* Fe_2_N and Fe^0^, implying that a conversion reaction possibly occurred in the Fe_2_N anode.^[^ ^34^, ^35^, ^36^ ^]^

When the anode returns to a fully charged state, only the peak of Fe_2_N was detected without other Fe‐based phases, indicating the excellent reversible electrochemical process. Notably, the ex situ HRTEM images and corresponding fast Fourier transform (FFT) patterns allow direct visualization of the Li‐ion intercalation and conversion process in Fe_2_N anode. At the fully lithiation state (0.01 V), the lattice fringe spacing of 0.174, 0.210 and 0.278 nm corresponded to the (103), (100) and (003) planes of Li_3_N, Fe and Li*_x_* Fe_2_N (Figure 3c,d), respectively, which further manifested the occurrence of partial conversion reaction.^[^ ^37^ ^]^ Upon the fully delithiation state (3.0 V), the lattice fringes with interplanar distances of 0.212 and 0.240 nm were consistent with the (102) and (021) planes of Fe_2_N (Figure 3e,f), demonstrating the extraction of Li‐ions from the electrode. In short, a multi‐faceted investigation has been carried out to identify the electrochemical reaction mechanism of Fe_2_N LIBs, and the results show that this is an indicative of a typical intercalation–conversion hybrid reaction, which is summarized in Figure 3g. This intercalation–conversion mechanism may highly enhance the structural stability, which directly correlates with electrochemical and kinetics performance. The corresponding charge storage reaction can be described as follows:

(4) $$2{\mathrm{Fe}}_{2}N+\left(x+3\right){\mathrm{Li}}^{+}+\left(x+3\right)e^{-}⇌{\mathrm{Li}}_{x}{\mathrm{Fe}}_{2}N+2\mathrm{Fe}+{\mathrm{Li}}_{3}N$$

**Figure 4:**
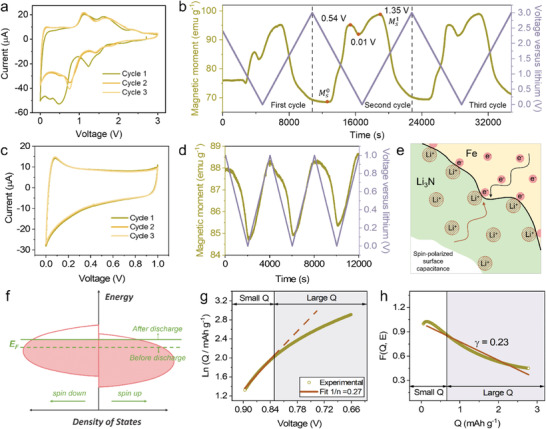
In situ magnetic characterization and thermodynamic analysis of the Fe_2_N anode. CV curves of a Fe_2_N electrode at a scan rate of 0.5 mV s^−1^ over the potential window of 0.01–3.0 V (a) and 0.01–1.0 V (c), respectively. b,d) Their corresponding in situ magnetic responses at an applied magnetic field of 3 T. e) Formation of a space charge zone in the surface capacitance model for extra lithium storage. f) Schematic of spin‐polarized density of states at the surface of ferromagnetic metal grains (before and after discharge). g) Dependence of heterogeneous storage on lithium activity in Fe:Li_3_N nanocomposites, indicating high (large Q) and low (small Q) storage regimes. The linear plot reveals a power law with the expected exponent. h) Dependence of *F*(*Q*, *E*) on Q in the heterogeneous storage process in the Fe:Li_3_N system.

Strikingly, in situ magnetometry is a highly sensitive and non‐invasive method for investigating the magnetic changes in real time.^[^ ^38^, ^39^ ^]^ To grasp clearer understanding of the reaction mechanism in Fe_2_N electrode, we carried out in situ magnetometry that was combined with the CV tests. **Figure** **4**a,b shows real time magnetic responses accompanying the electrochemically‐driven reactions with first three cycles. Clearly, the Fe_2_N electrode magnetization response in the first discharge–charge is different from the other cycles, which is due to the irreversible phase transformation of Fe_2_N during the first lithiation/delithiation. When the Fe_2_N LIBs discharge from 3.0 to 0.54 V in the second cycle, the magnetization (*M_S_*) exhibits a sharp increase up to 95.1 emu g^−1^, which is attributed to the reduction of Fe_2_N to metallic Fe^0^. Subsequently, the *M*_*S*_ decreases to a value of 91.8 emu g^−1^ at the end of the discharge, which can be reminiscent of our recently proposed spin‐polarized surface capacitance behavior.^[^ ^40^, ^41^, ^42^ ^]^ To further verify the finding, in situ magnetometry was performed upon cycling at low voltage (to exclude the effect of redox) between 0.01 and 1.0 V. As shown in Figure 4c, these curves exhibit a more rectangle shaped profile, indicating a pseudocapacitive behavior. Meanwhile, the *M*_*S*_ rises as the voltage goes up and falls when the voltage goes down (Figure 4d) in this scanning domain, which exactly corresponds to the spin‐polarized surface capacitance phenomenon. This phenomenon can be reasonably explained by the fact that the reduction product Fe^0^ nanoparticles possess a high density of states (DOS) at the Fermi level due to the highly localized *d*‐orbitals. Accordingly, electrons accumulate inside the surface of reduced Fe^0^ nanoparticles within a Thomas–Fermi screening length whereas lithium ions are stored at the grain boundaries and surfaces^[^ ^41^ ^]^ (Figure 4e). Figure 4f shows the density of states (DOS) at the surface of Fe^0^ nanoparticles before and after discharge. Specifically, the accumulation of electrons in the spin‐up bands are more than that in the spin‐down bands owing to the spin‐minority states being dominant near the Fermi level on the surface of 3*d* ferromagnetic metals, which demonstrates that the *M*_*S*_ will show a downward trend because the extra electrons preferentially fill the spin‐down bands during the last stage of discharge. Inversely, a pronounced increase in the magnetization reaching 98.8 emu g^−1^ is observed when the voltage increases to 1.35 V, which could be interpreted as the release of spin‐polarized electrons. On further charge, the *M*_*S*_ shows a monotonic decrease to 69.4 emu g^−1^ because of the oxidation of Fe^0^ to Fe_2_N. Moreover, the conversion ratio can be roughly quantified through comparing the Δ*M*_*S*_ to its theoretical value $\Delta {M^{\prime }}_{S}$. To avoid the effect of spin‐polarized surface capacitance, the net magnetic moment Δ*M*_*S*_ was used for calculation (Δ*M*_*S*_ = $M_{S}^{1}-M_{S}^{0}$, ca. 30.4 emu g^−1^ as exhibited in Figure 4a). Based on the previous report^[^ ^43^ ^]^ that the *M*_*S*_ of Fe is 217.0 emu g^−1^, and the theoretical value $\Delta {M^{\prime }}_{S}$ should be 192.9 emu g^−1^ for the complete conversion of Fe_2_N to Fe^0^. Consequently, the ratio of the part where conversion reaction occurs to the entire active material can be estimated to be 24.4%.

In fact, the spin‐polarized surface capacitance observed in this work is consistent with the “job‐sharing” mechanism‐based heterogeneous storage proposed by Maier on principle.^[^ ^44^, ^45^, ^46^ ^]^ This behavior allows for fast storage, because Li^+^ transport along one phase and e^−^ transport along the other. We will now use the thermodynamic analysis to further investigate this charge storage behavior. As depicted in Figure S8, Supporting Information, the related discharge curve shows that Fe_2_N goes through different storage modes:^[^ ^44^ ^]^ single phase storage in Fe_2_N (I); two‐phase storage by forming Li*_x_* Fe_2_N (II); conversion to Fe:Li_3_N (III); and the heterogeneous storage (IV). Based on numerous assumptions and calculations, Maier’ group concluded that in the case of satisfying the heterogeneous storage reaction, the relationship between capacity (*Q*) and voltage (*E*) could be formulated as:

(5) $$\exp\left(-\frac{\mathrm{eE}}{k_{B}T}\right)\propto a_{\mathrm{Li}}\propto Q^{n}\exp\left(\gamma Q\right)$$

The Equation (5) can be further simplified to:

(6) $$E+n\frac{k_{B}T}{e}\ln Q=-\gamma \frac{k_{B}T}{e}Q-\frac{k_{B}T}{e}\ln k$$

(7) $$\ln Q=-\frac{e}{nk_{B}T}E-\frac{\gamma Q}{n}-\frac{\ln k}{n}$$

where *k*_B_ is the Boltzmann’ constant, *T* is the thermodynamic temperature, *a*_Li_ is the Li activity, and *n* is the concentration of conduction electrons. According to Equation (7), we analyzed the linear relationship between *lnQ* and *E* presented in Figure 4g, and fitted *n* value is ≈3.7, which indicates that the whole curve linearizes very nicely with slopes of the expected magnitude (3 < *n* < 4).^[^ ^44^ ^]^ Subsequently, in Equation (6) let:

(8) $$F\left(E,Q\right)=E+n\frac{k_{B}T}{e}\ln Q$$

then

(9) $$F\left(E,Q\right)=-\gamma \frac{k_{B}T}{e}Q-\frac{k_{B}T}{e}\ln k$$

where *γ* value represents the expected order of magnitude for the whole curve linearizes and to be roughly on the order of 1 g (mAh)^−1^. Substituted the value of *n* into Equation (8) and combined Equation (9), we could obtain an excellent linear relationship between *F*(*E*, *Q*) and *Q*, as illustrated in Figure 4h, the final fitted value of *γ* is 0.23 g (mAh)^−1^. Taken together, the thermodynamic calculation results confirm the existence of a “job‐sharing” mechanism‐based heterogeneous storage with fast kinetic response during the Li‐ion storage process, which may be one of the reasons for the good kinetic properties of Fe_2_N materials. At this point the reaction mechanism of Fe_2_N can be described as an intercalation–conversion–heterogeneity hybrid storage. Therefore, in addition to Equation (4), the following Equation (10) can occur:

(10) $$\mathrm{Fe}/{\mathrm{Li}}_{3}N+n{\mathrm{Li}}^{+}+ne^{-}↔{\mathrm{Fe}}^{n-}/n{\mathrm{Li}}^{+}/{\mathrm{Li}}_{3}N$$

**Figure 5:**
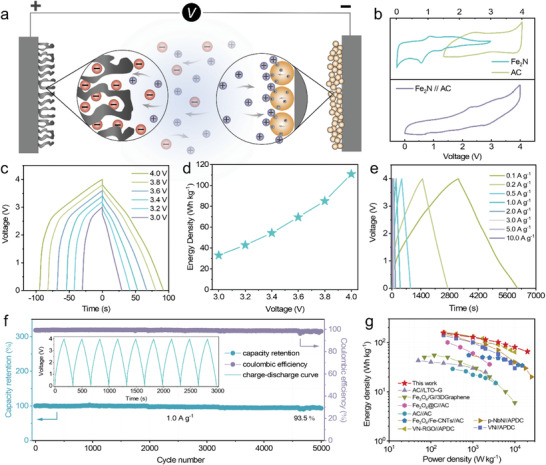
Electrochemical evaluation of the Fe_2_N//AC LICs. a) Schematic illustration of the Fe_2_N//AC LICs device. b) CV profiles of the Fe_2_N and the AC electrodes in half cells (top) and full cell of LICs (bottom) at 0.5 mV s^−1^. c) Galvanostatic charge/discharge voltage profiles at different voltage windows (at fixed 0.2 A g^−1^). d) Energy density of the LICs with the increase in the voltage window at a fixed current density of 0.2 A g^−1^. e) Galvanostatic charge/discharge voltage profiles at different current density between 0.01 and 4.0 V. f) Cycle performance and Coulombic efficiency for 5000 cycles at a current density of 1.0 A g^−1^. g) Ragone plots for the Fe_2_N//AC LICs based on the total mass of active material in both electrodes and compared with other LICs reported in the literature.^[^ ^49^, ^50^, ^51^, ^52^, ^53^, ^54^ ^]^

In the end, an asymmetric full‐cell LICs, denoted as Fe_2_N//AC, was assembled using the Fe_2_N as the anode and commercial AC as the cathode, as is schematically shown in **Figure** **5**a. This AC material has advantages that make it suitable for energy storage applications, such as good electrical conductivity, large surface areas and tunable porosity. Before assembling the full‐cells, the electrochemical performance of the half‐cells with AC as the cathode and metallic lithium as the anode was performed, as shown in Figure S9, Supporting Information. Upon charging, the Li^+^ cations are intercalated into the Fe_2_N anode along with the ${\mathrm{PF}}_{6}^{-}$ anions are absorbed at the surface of AC cathode. The discharge process of the LICs is vice versa. Here, in terms of Fe_2_N anode and AC cathode, the optimal mass ratio between the two electrodes was determined in the LIC on a basis of the charge balance theory (*q* ^+^ = *q* ^−^).^[^ ^47^, ^48^ ^]^ To determine the optimal operating voltage window of thus‐constructed Fe_2_N//AC full‐cell, the voltage range of individual anode and cathode was first probed by CV test in half‐cell configuration (Figure 5b, upper panel). Moreover, a series of CV tests of Fe_2_N//AC at different voltage windows were implemented as depicted in Figure S10, Supporting Information. Of note, the acquisition of Faradaic reactions increases as the operating potential window increases to 4.0 V, whereas serious limitations for the side reaction are observed when the voltage is greater than or equal to 4.2 V. The GCD curves for this device at different voltage window indicate the discharge time increased strikingly with the increase in the voltage window from 3.0 to 4.0 V (Figure 5c). And the corresponding energy density was remarkably improved from 33.0 to 110.9 Wh kg^−1^ (Figure 5d). Accordingly, we chose a potential window of 0.01–4.0 V to further evaluate the electrochemical performance for the LICs.

As depicted in Figure 5e, the GCD curves of the LIC at different current densities from 0.1 to 10.0 A g^−1^ exhibit symmetric quasi‐triangular shapes, substantiating the combination of Faradaic and non‐Faradaic processes. The cycling stability of the Fe_2_N//AC LICs (Figure 5f) was investigated at the current density of 1.0 A g^−1^, and the device maintains its Coulombic efficiency of nearly 100% up to 5000 cycles with an energy density retention of 93.5%. In addition, the high rate performance is evidenced in the Ragone plots (Figure 5g), showing the trade‐off between energy and power density of the present Fe_2_N//AC LICs. Specifically, the device can deliver an energy density up to 157 Wh kg^−1^ at a power density of 200 W kg^−1^, and it still maintains an impressive energy density of 65 Wh kg^−1^ even at power outputs as high as 20 000 W kg^−1^.

## 3 Conclusion

In summary, we investigated a typical TMN electrode material, 3D porous Fe_2_N micro‐coral, with a fast rate capability and high energy density. Most importantly, the Li‐ion storage mechanism is comprehensively investigated by a series of characterization techniques and thermodynamic analysis, and the result reveals that the storage mechanism involves an intercalation–conversion–heterogeneity hybrid reaction. We also show that the magnetic response during charge–discharge process in real time is capable of quantifying the ratio (ca. 24.4%) of the part where conversion reaction occurs to the entire active material. Noticeably, a novel LIC device based on the as‐prepared Fe_2_N anode and AC cathode also delivers an outstanding power density of 4000 W kg^−1^ at a high energy density of 100.7 Wh kg^−1^ as well as excellent capacity retention property.

## 4 Experimental Section

### Material Synthesis

Typically, 1 g ferric chloride hexahydrate (FeCl_3_·6H_2_O, 99.9%, Aladdin), 2.4 g sodium citrate dihydrate (Na_3_C_6_H_5_O_7_·2H_2_O, 99.9%, Merck), 0.8 g urea (CH_4_N_2_O, 99.9%, Merck), and 0.6 g sodium polyacrylate ((C_3_H_3_NaO_2_)n, *M*_w_ = 3 000 000–7 000 000, Aladdin) were dissolved in 80 mL distilled water by vigorously stirring for 2 h to form a homogeneous solution. Then, the mixture was transferred to a 100 mL Teflon‐lined stainless steel autoclave and maintained at 200 °C for 4 h. After the reaction was complete, the black precipitate was collected by centrifugation and washed three times using deionized water and ethanol, followed by drying at 60 °C under vacuum overnight. The dried sample was grinded and calcined in Ar atmosphere at 400 °C for 4 h to obtain Fe_3_O_4_ nanosphere precursors. By further applying thermal anneal at 800 °C for 2 h with a heating rate of 5 °C min^−1^ under NH_3_ atmosphere, 3D porous Fe_2_N micro‐corals could be obtained.

### Material Characterization

The crystalline structures of the sample were characterized using X‐ray diffraction (Bruker D8 Advance Diffractometer) with a Cu K*α* radiation. The morphology and structure of the samples were inspected by the scanning electron microscopy (SEM, ZEISS, Sigma 300) and TEM (JEOL, JEM‐2100F). To investigate the surface chemical composition and valent states of the products, the XPS (ESCALAB250Xi) was employed. The evolution of phase and crystallinity of the Fe_2_N during the charging and discharging process were detected by using a specially designed in situ XRD electrochemical cell that was positioned within a Rigaku Ultima IV diffractometer with Cu K*α* radiation (*λ* = 1.5046 Å). The whole assembly process was done in an argon‐filled glovebox, and the applied current was 200 mA g^−1^ with the 2*θ* values ranging from 20° to 70°.

### Electrochemical Measurements

The working electrodes were prepared by coating a mixture of active materials, super‐P carbon, and carboxymethyl cellulose (CMC) binder with a weight ratio of 7:2:1 on a copper foil. The 2032‐type coin cells (CR2032) were assembled with lithium metal as counter and reference electrode in an Ar‐filled glove box ([O_2_] < 0.01 ppm, [H_2_O] < 0.01 ppm). For the lithium‐ions half‐cells, 1.0 m solution of LiPF_6_ dissolved in a 1/1 (w/w) mixture of ethylene carbonate (EC) and diethyl carbonate (DEC) was used as the electrolyte, and the Celgard 2250 film (Whatman) as the separators. Galvanostatic charge–discharge tests were obtained on a Landt CT2001A test system between 0.01 and 3.0 V versus Li^+^/Li. The CV at various scan rates and electrochemical impedance spectroscopy (EIS) were carried out at an electrochemical workstation (IVIUM technologies, Vetex). For the full‐cells, the Fe_2_N electrode and AC (Kurerey Chemical Company) electrode were used as anode and cathode, respectively, while the separator and electrolyte were the same with the above half‐cells. Before assembling the capacitor, the two electrodes were activated as three cycles in half batteries at 0.2 A g^−1^. The electrochemical performances of the capacitors were tested with the potential window between 0.01 and 4.0 V. The energy (E) and power densities (P) of the LICs were calculated based on the total mass of both anode and cathode materials according to the following equations:

(11) $$E=\int_{t_{2}}^{t_{1}}IV\;dt=\frac{1}{2}C\left(V_{\max}+V_{\min}\right)\left(V_{\max}-V_{\min}\right)=\Delta V\times \frac{I}{m}\times t$$

(12) $$P=\frac{E}{t}=\Delta V\times \frac{I}{m}$$

(13) $$\Delta V=\frac{\left(V_{\max}+V_{\min}\right)}{2}$$

where *I* is the charge/discharge current; *m* is the total mass of the both electrodes; *V*_min_ and *V*_max_ are the potential at the beginning and the end of the charge; *C* is the capacitance of the LICs.

### In Situ Magnetometry

The LIBs for the in situ magnetometry test were assembled with Fe_2_N cathode, lithium foil anode, and electrolyte in the argon‐filled glove box at room temperature. To facilitate the in situ magnetization test, the cells were sealed with the flexible polyethylene terephthalate sheets. The magnetic properties were probed by a Quantum Design physical property measurement system (PPMS) magnetometer at 300 K. The Fe_2_N LIBs were connected in galvanostatic mode at 200 mA g^−1^ with the potential window between 0.01 and 3.0 V. All in situ magnetization measurements were carried out simultaneously with the electrochemical discharge–charge processes in magnetic fields that were parallel to the copper foil.

## Conflict of Interest

The authors declare no conflict of interest.

## Supporting information

Supporting Information

## Data Availability

The data that support the findings of this study are available from the corresponding author upon reasonable request.
