# Supplementary material for: Revealing An Intercalation‐Conversion‐Heterogeneity Hybrid Lithium‐Ion Storage Mechanism in Transition Metal Nitrides Electrodes with Jointly Fast Charging Capability and High Energy Output
Source: Adv Sci (Weinh). 2022 Oct 6;9(33):2203895. doi: 10.1002/advs.202203895 (PMC9685454; doi:10.1002/advs.202203895)
Supplement: Supplementary file 1 — Supporting Information [file ADVS-9-2203895-s001.pdf]

ADVANCED SCIENCE
Open Access

Supporting Information

for *Adv. Sci.*, DOI 10.1002/advs.202203895

Revealing An Intercalation-Conversion-Heterogeneity Hybrid Lithium-Ion Storage Mechanism in Transition Metal Nitrides Electrodes with Jointly Fast Charging Capability and High Energy Output

*Fei Li, Yadong Li, Linyi Zhao, Jie Liu, Fengkai Zuo, Fangchao Gu, Hengjun Liu, Renbin Liu, Yuhao Li, Jiqiang Zhan, Qiang Li and Hongsen Li**

Supporting Information

Revealing an Intercalation-Conversion-Heterogeneity Hybrid Lithium-Ion Storage Mechanism in Transition Metal Nitrides Electrodes with Jointly Fast Charging Capability and High Energy Output

*Fei Li,[§] Yadong Li,[§] Linyi Zhao, Jie Liu, Fengkai Zuo, Fangchao Gu, Hengjun Liu, Renbin Liu, Yuhao Li, Jiqiang Zhan, Qiang Li, and Hongsen Li**

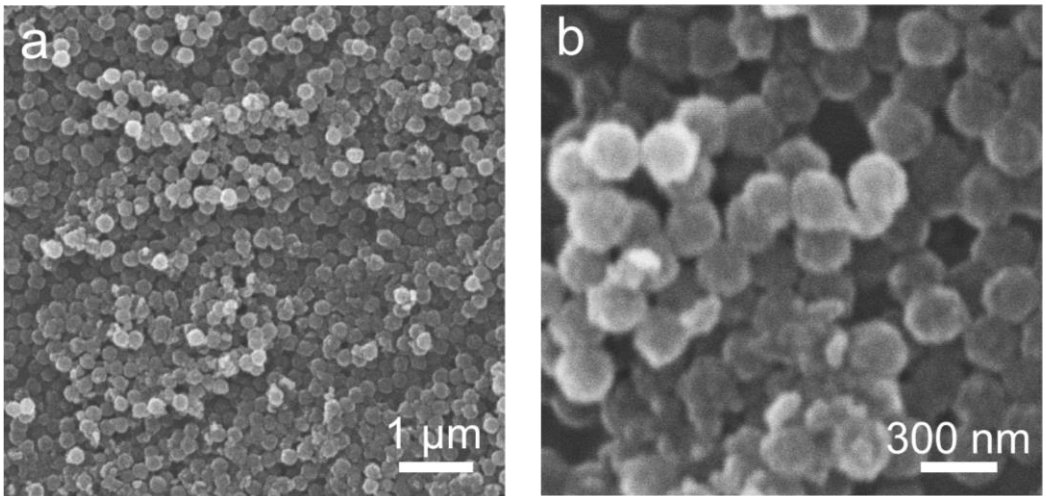

Figure S1. SEM images of the Fe_3O_4 nanospheres.

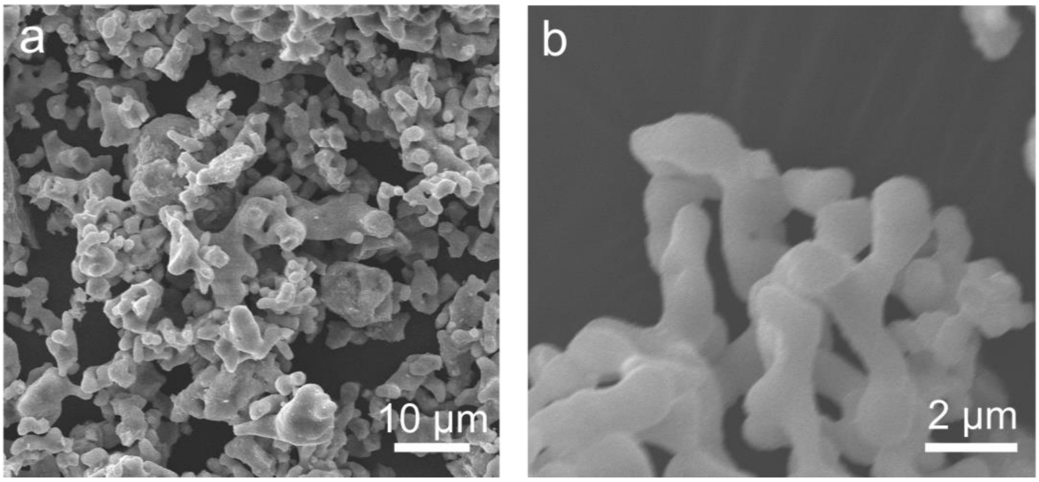

Figure S2. SEM images of the as-prepared 3D porous Fe_2N micro-corals.

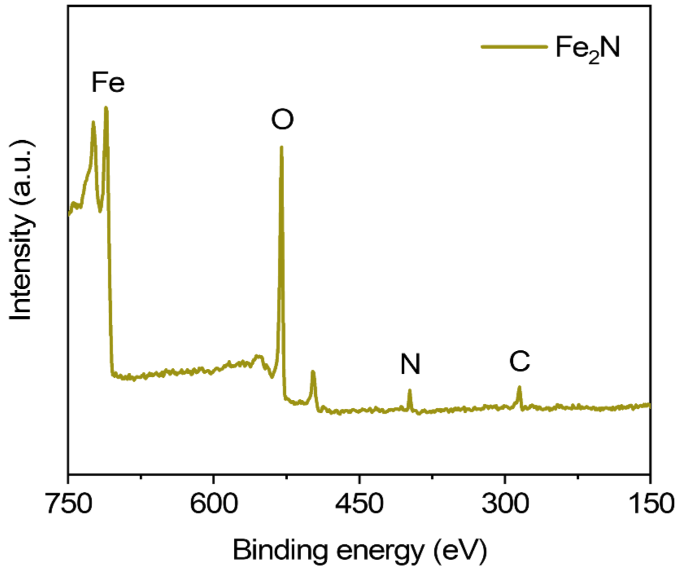

Figure S3. The survey spectrum of Fe_2N

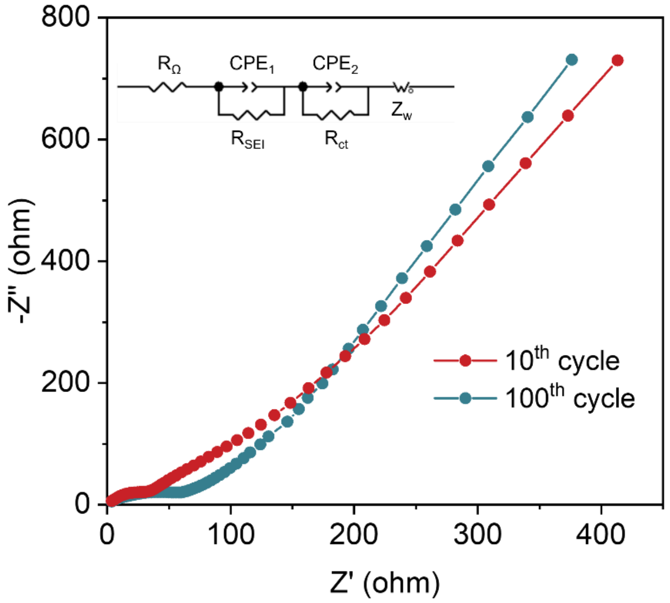

Figure S4. Nyquist plots of the Fe_2N electrodes at different electrochemical states (inset: selected equivalent circuit).

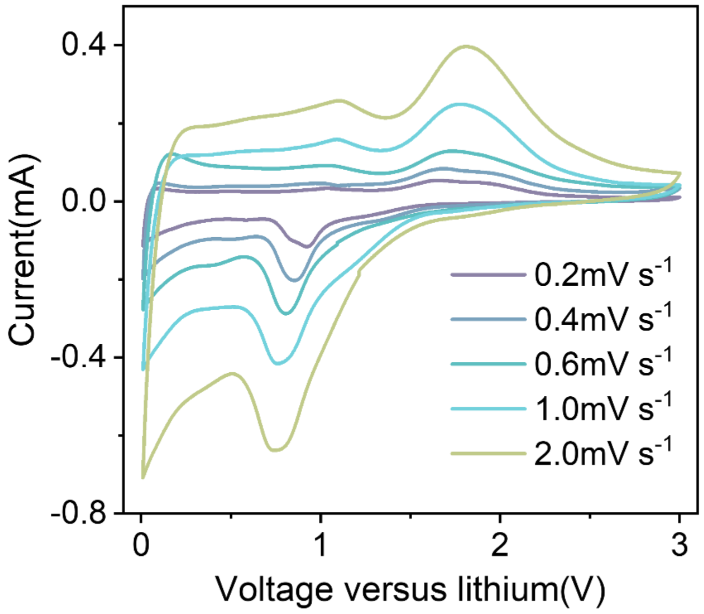

Figure S5. The CVs of the Fe_2N electrode at different scan rates from 0.2 to 2.0 mV s^{-1}.

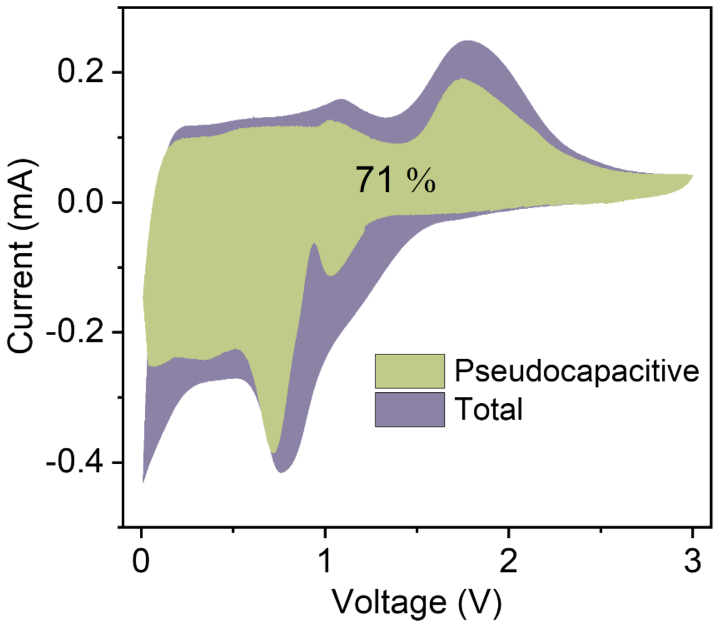

Figure S6. Capacitive and diffusion-controlled contribution to charge storage at a scan rate of 0.5 mV s^{-1}. The capacitive current is shaded and compared with the total measured current.

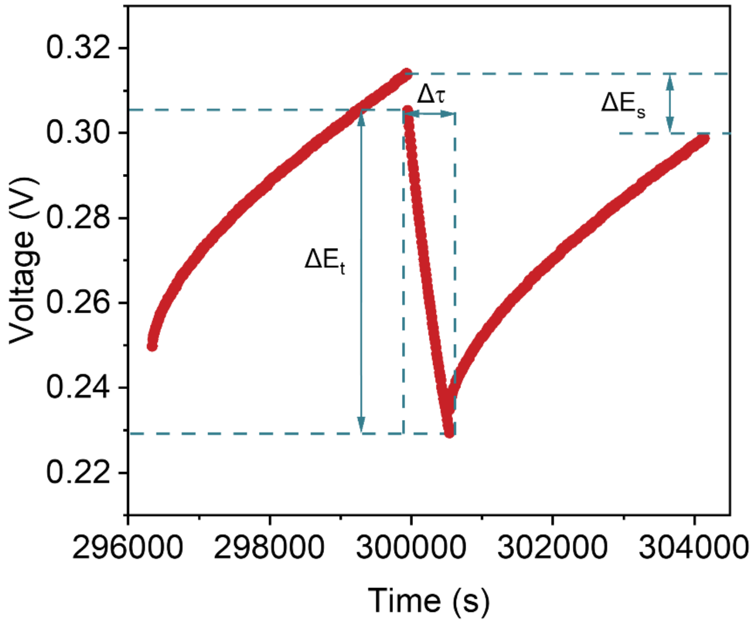

Figure S7. An enlarged view of the Fe_2N half-cell potential during a GITT pulse.

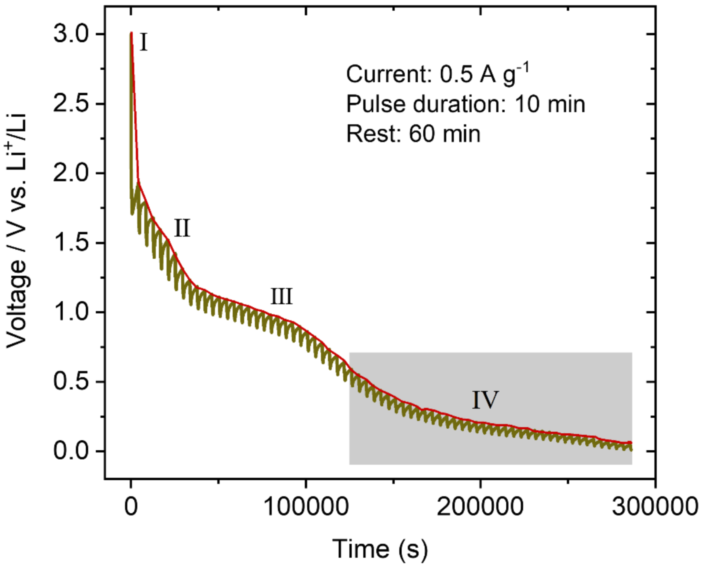

Figure S8. The open-circuit voltage of the Fe_2N half-cell as a function of time was obtained by GITT.

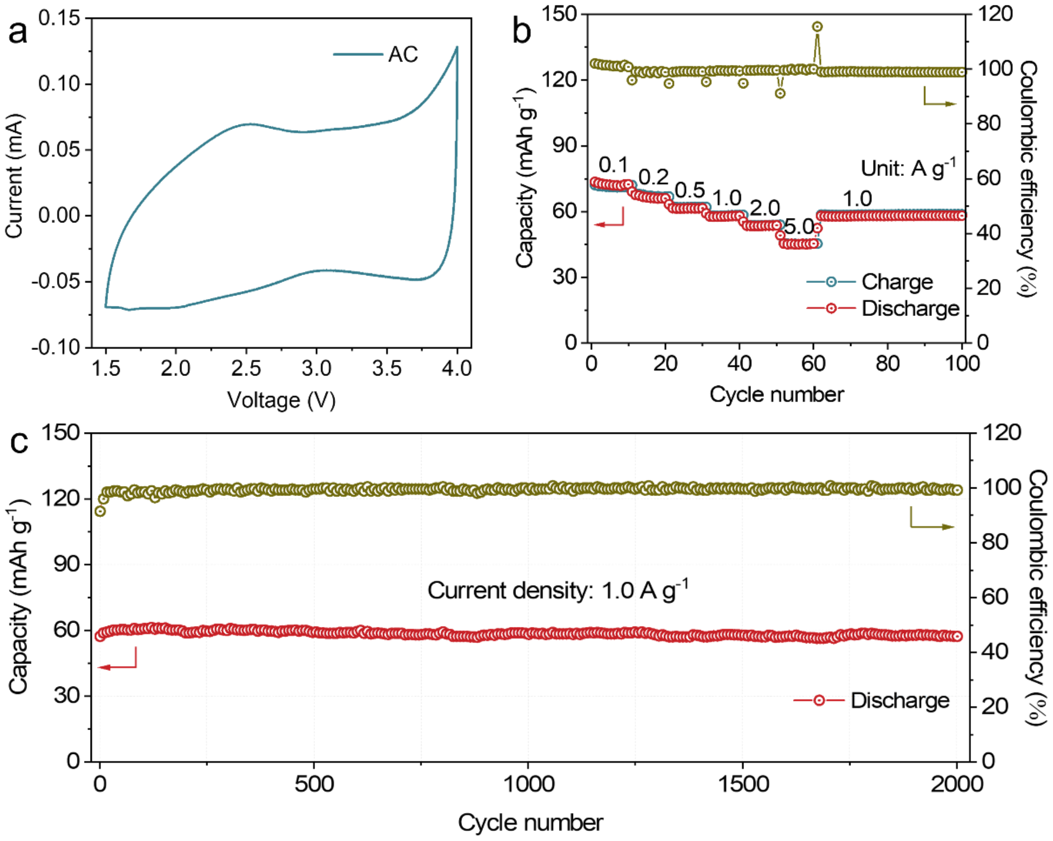

Figure S9. Electrochemical analysis of AC electrode in half-cells. a, CV curves at 0.5 mV s^{-1}; **b,** rate performance at various current densities; **c,** cyclic performance at 1.0 A g^{-1}.

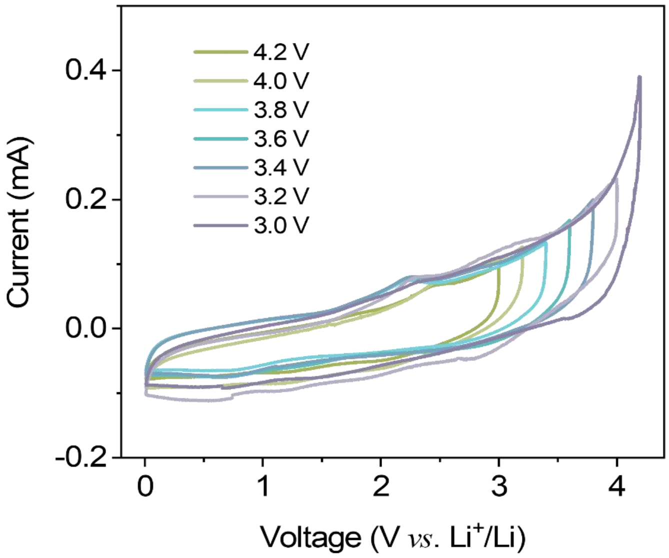

Figure S10. CV curves of the LICs at different voltage windows at a scan rate of 0.5 mV s^{-1}.

Table S1. Impedance parameters, R_e and R_{ct} of Fe_2N samples with different cycles.

Parameter	10th	100th
R_e (Ω)	3.707	6.611
R_{ct} (Ω)	49.25	72.37
